# Kaolin-Derived Porous Silico-Aluminate Nanoparticles as Absorbents for Emergency Disposal of Toluene Leakage

**DOI:** 10.3390/molecules29112624

**Published:** 2024-06-03

**Authors:** Xin Wang, Guishi Rao, Feng Zhou, Fuli Bian, Yuan Hu

**Affiliations:** 1 State Key Laboratory of Fire Science, University of Science and Technology of China, No. 96, Jinzhai Road, Hefei 230026, China; 2 Jiangxi Academy of Emergency Management Science, No. 1519, Chuntai Road, Xinjian District, Nanchang 330199, China; 3 Shanghai Fire Research Institute of Ministry of Emergency Management of China, No. 601, Zhongshan South 2nd Road, Xuhui District, Shanghai 200032, China; 4 Shanghai Qiangshi Fire Equipment Co., Ltd., No. 1100, Linxian Road, Jinshan District, Shanghai 201505, China

**Keywords:** silico-aluminate nanoparticles, absorbents, emergency disposal, toluene leakage

## Abstract

To rapidly eliminate toluene from aqueous environments during leakage accidents, this paper synthesized porous silico-aluminate nanoparticles (SANs) via a hydrothermal method, using cost-effective and non-toxic natural kaolin as the basic raw material. The morphology and structure of the porous SANs were characterized using scanning electron microscopy (SEM), X-ray photoelectron spectroscopy (XPS), X-ray diffraction (XRD), and BET-specific surface area tests. The effects of different conditions, such as the dosage of porous SANs, initial concentration of toluene, temperature, capture time, and pH, on the adsorption performance of porous SANs were analyzed. The as-prepared SANs exhibited a high removal efficiency and rapid adsorption performance toward toluene in aqueous solution. Finally, the kinetics of the adsorption of toluene in aqueous solution by porous SANs were investigated. The mechanism of the adsorption of toluene by porous SANs was further discussed. These findings provide a cost-effective and highly efficient absorbent for the emergency disposal of toluene leakage accidents.

## 1. Introduction

Toluene, an important basic chemical material, has been widely used in the production of pharmaceutical and pesticide intermediates such as trifluoromethylene, m-nitrotrifluoromethylene, and p-chlorotrifluoromethylene in the fluorine chemical industry [1]. However, due to the high toxicity of toluene, once it leaks into water, it deteriorates the aqueous environment and is difficult to remove through microbial metabolism and degradation in the water. Toluene leakage causes long-term and sustained negative impacts on aquatic organisms, endangering human health and damaging the ecological environment [2,3].

To address the hazardous chemical leakage, various approaches, such as physical adsorption, in-situ combustion, biodegradation, mechanical recovery, and filtration membrane methods, have been adopted [4,5,6,7,8]. Among these approaches, the use of adsorbents to adsorb leaked hazardous chemicals has many advantages, such as simple and convenient operation, high recovery efficiency, and environmental protection [9,10,11], and it plays an irreplaceable role in the emergency disposal of hazardous liquid chemical spills. Therefore, the development of high-performance adsorbents for the emergency disposal of hazardous chemical spills has received widespread attention in recent years [12]. Generally, porous natural materials with large specific surface areas are chosen for adsorption applications. Natural adsorbents include activated carbon, bentonite, vermiculite, sawdust, straw, rice husk, sugarcane bagasse, etc. [12,13,14,15,16,17,18]. Although natural adsorbents have the advantages of abundant sources and low cost, their weak adsorption capacity and poor oil–water selectivity limit their widespread application.

To overcome the above shortcomings, many modified adsorbents based on natural materials have been developed that effectively improve the adsorption performance of products to a certain extent [19,20,21,22]. Moura et al. successfully synthesized carbon nanofibers and carbon nanotubes on the surface of vermiculite using a chemical vapor deposition method and prepared a sponge-like vermiculite-based adsorbent. The results showed that the adsorption capacity for oil significantly improved and was six times higher than that of unmodified adsorbents [23]. Deschamps et al. modified the surface of cotton fibers using fatty acids under microwave radiation. The results showed that the amount of oil absorbed was approximately 20 times its own weight. The oil–water selectivity of modified cotton fibers was greatly improved, and the acetylated cotton fibers also showed superior stability [24]. Kenes et al. pyrolyzed rice husks at 700 °C to produce amorphous silica. Adsorption tests showed that their adsorption capacity for heavy oil could reach 15 times their own weight [25]. Angelova et al. prepared C/SiO_2_ porous composite materials via the pyrolysis of rice husks at 480 °C. Adsorption kinetics study showed that the adsorption capacity was closely related to the particle size composition and volume density [26]. Compared with natural adsorbents, modified adsorbents have many advantages, such as high saturation adsorption capacity, strong adsorption retention capacity, and high oil–water selectivity, and have good application prospects in the field of emergency disposal of hazardous chemical spills.

As a type of silico-aluminate crystal material with a special pore structure, zeolite molecular sieves not only have advantages such as a large specific surface area, good stability, and adjustable porosity but their hydrophilicity (hydrophobicity) and structural characteristics can also be controlled by adjusting the silicon aluminum ratio [27,28,29]. Therefore, zeolite molecular sieves have been widely used in the chemical industry as adsorbents [30]. At present, various zeolite molecular sieves are often used as selective adsorbents for the adsorption and separation of gas organic molecules [31,32,33]. However, due to factors such as economic cost and processing, the application of zeolite molecular sieves in the emergency disposal of organic pollutants in water has rarely been reported.

In this paper, porous silico-aluminate nanoparticles were synthesized via a hydrothermal method, using inexpensive and non-toxic natural kaolin as the basic raw material. The morphology and structure of the porous silico-aluminate nanoparticles were characterized using SEM, XPS, XRD, and BET-specific surface area tests. The effects of various conditions, such as the amount of porous silico-aluminate nanoparticles, initial toluene concentration, temperature, capture time, and pH, on the adsorption performance were investigated. The adsorption kinetics were also analyzed to reveal the adsorption mechanism of toluene by the porous silico-aluminate nanoparticles.

## 2. Materials and Methods

### 2.1. Materials

Kaolin was provided by Xinglu Clay Mine Co., Ltd. (Jiujiang, China). Toluene, sulfuric acid, sodium silicate, hexadecyl trimethyl ammonium bromide, and sodium hydroxide were analytic grade and purchased from Aladdin Scientific Corp. (Shanghai, China).

### 2.2. Preparation of Porous Silico-Aluminate Nanoparticles

The specific preparation process for silico-aluminate nanoparticles included the following three main stages: (1) natural kaolin was crushed and calcined in a muffle furnace at 800 °C for 6 h to obtain metakaolin. (2) A certain amount of kaolin and an aqueous solution of sodium hydroxide (5 mol·L^−1^) were added to a beaker. The mixture was stirred continuously to obtain a uniform slurry and then transferred to a round bottom flask. Then, a certain amount of sodium silicate was added to the above mixture, and the mixture was heated to 98 °C for 2 h with stirring at 600 r·min^−1^. The mixture was kept still and cooled to room temperature to obtain zeolite nanoclusters containing Y-type secondary structures. (3) An aqueous solution of cetyltrimethylammonium bromide (CTAB) (0.5 mol·L^−1^) was slowly added to the above zeolite nanocluster sol, and then the mixture was stirred quickly for 10 min. The pH of the mixture was adjusted to 11 with sulfuric acid via ultrasonication (frequency of 50 kHz, power of 1000 W), and the mixture was subsequently heated in a microwave oven (frequency of 2000 MHz, power of 1000 W) for 100 min. The mixture was then kept at 90–100 °C for static crystallization. After cooling to room temperature, the mixture was filtered, and the filter cake was washed with deionized water. After drying at 80 °C, the porous silico-aluminate nanoparticles were obtained by roasting in a muffle furnace at 600 °C for 6 h.

### 2.3. Experimental Method for Capturing Toluene in Porous Silico-Aluminate Nanoparticles

#### 2.3.1. Effects of Different Dosages

A set of 100 mL iodine bottles was taken and 25 mL of toluene aqueous solution with a concentration of 20 mg·L^−1^ that was prepared in advance was added. Then, 0.1 g, 0.2 g, 0.3 g, 0.4 g, 0.5 g, 0.6 g, 0.8 g, and 1.0 g porous silica-aluminum nanomaterials were added into each iodine bottle successively and the stoppers covered tightly. After oscillating in an oscillator at 20 °C and 150 r·min^−1^ for 120 min, the system was transferred to a centrifuge tube for centrifugation and filtration, and the equilibrium concentration of toluene residue in the filtrate after capture was determined using UV-vis spectrophotometry. The above experiment was repeated three times and the average value taken. The rates of adsorption of dissolved toluene in aqueous solution by porous silica-Al nanomaterials with different dosages were calculated.

#### 2.3.2. Effect of the Capture Time

A set of 100 mL iodine vials was taken and 25 mL of pre-prepared toluene aqueous solution with a concentration of 20 mg·L^−1^ was added. Then, 0.6 g of porous silicon and aluminum nanomaterials was added into each iodine vial, and the stopper covered tightly. The vials were oscillated in the oscillator at 20 °C and 150 r·min^−1^ for 5 min, 10 min, 20 min, 30 min, 40 min, 50 min, 60 min, 80 min, 120 min, and 180 min, and then transferred to the centrifuge tube for centrifugation and filtration. Uv-vis spectrophotometry was used to determine the equilibrium concentration of toluene residue in the filtrate after capture and elimination. The above experiment was repeated three times and the average value taken. The rates of adsorption of dissolved toluene in aqueous solution by porous silica-Al nanomaterials under different trapping times were calculated.

#### 2.3.3. Effect of pH

A set of 100 mL iodine vials was taken and 25 mL of toluene aqueous solution with pH values of 2, 4, 6, 8, 10, and 12 and a concentration of 20 mg·L^−1^ were added. Then, 0.6 g of porous silica-aluminum nanomaterials was added into each iodine vial and covered with a stopper. The equilibrium concentration of toluene residue in the filtrate after capture was determined using Uv-vis spectrophotometry after 120 min of oscillation at 25 °C and 150 r·min^−1^ in the oscillator. The above experiment was repeated three times and the average value taken. Then, the toluene capture and elimination rates of porous silica-Al nanomaterials at different pH values were calculated. The pH of the solution under acidic conditions was adjusted using sulfuric acid, and the pH of the solution under alkaline conditions was adjusted using sodium hydroxide.

#### 2.3.4. Effect of Temperature

A set of 100 mL iodine vials was taken and 25 mL of pre-prepared toluene aqueous solution with a concentration of 20 mg·L^−1^ was added. Then, 0.6 g of porous silicon and aluminum nanomaterials was added into each iodine vial, and the stopper covered tightly. The vials were then oscillated in an oscillator at 0 °C, 10 °C, 20 °C, 30 °C, 40 °C, and 150 r·min^−1^ for 120 min, transferred to a centrifuge tube for centrifugation and filtration. The equilibrium concentration of toluene residue in the filtrate after capture was determined using UV-vis spectrophotometry. The above experiment was repeated three times and the average value taken. The rates of adsorption of dissolved toluene in aqueous solution by porous silica-Al nanomaterials under different trapping times were calculated.

#### 2.3.5. Kinetic Study Methods

A set of 100 mL iodine bottles were taken and divided into three groups. Into the first group was added 25 mL of pre-prepared toluene water solution with a concentration of 20 mg·L^−1^, into the second group was added 25 mL of pre-prepared toluene water solution with a concentration of 50 mg·L^−1^, and into the third group was added 25 mL of pre-prepared toluene water solution with a concentration of 100 mg·L^−1^. Then, 0.6 g of porous silica-aluminum nanomaterial was added into each iodine measuring bottle and the plugs closed. The bottles were oscillated in the oscillator at 20 °C and 150 r·min^−1^ for 5 min, 10 min, 20 min, 30 min, 40 min, 50 min, 60 min, 80 min, 120 min, and 180 min, and transferred to a centrifuge tube for centrifugation and filtration. Uv-vis spectrophotometry was used to determine the equilibrium concentration of toluene residue in the filtrate after capture and elimination. The above experiment was repeated three times and the average value taken. Then, the adsorption capacities of porous silica-Al nanomaterials for dissolved toluene in aqueous solution were calculated under different trapping times.

### 2.4. Determination of Toluene Concentration in Aqueous Solution

The concentration of toluene in aqueous solution was determined using UV-vis spectrophotometry. First, the UV-vis spectrophotometer was used to scan the toluene solution at a certain wavelength range, and the maximum absorption wavelength λ_max_ was determined. Then, a series of toluene standard solutions with a certain concentration gradient was prepared, and their absorbance was measured at the maximum absorption wavelength λ_max_, and the standard curve of toluene solution was drawn according to the data on the corresponding relationship between absorbance and concentration. Finally, the absorbance of the toluene solution was measured at the maximum absorption wavelength λ_max_, and the corresponding concentration of the toluene solution was found using the standard curve.

### 2.5. Characterization

The morphology of the samples was observed using an LEO l530 scanning electron microscope (LEO, Berlin, Germany) at an accelerating voltage of 10 kV.

The specific surface area and pore structures were measured using an ASAP 2020 microporous physical adsorption and chemical adsorption analyzer (Micromeritics, Norcross, GA, USA).

The X-ray diffraction patterns of the samples were measured using a MiniFlex 600 X-ray diffractometer (Rigaku, Tokyo, Japan) with a Cu Kα (λ = 1.540 Å) laser source.

The chemical composition of the samples was measured using an ESCALAB 250Xi X-ray photoelectron spectrometer (Thermo Fisher, Waltham, MA, USA) with an Al Kα (hv = 1486.6 eV) excitation source.

The concentration of toluene in the aqueous solution was determined using UV–2100 UV-visible spectrophotometry (Shimadzu, Tokyo, Japan).

### 2.6. Calculation of the Adsorption Rate of Toluene in Aqueous Solution

The adsorption rate (η) of toluene in aqueous solution can be calculated using the following equation:

H = (C_0_ − C_e_)/C_0_, (1)

where C_0_ is the initial concentration of toluene in aqueous solution (mg·L^−1^), and Ce is the equilibrium concentration of residual toluene in the captured and disinfected aqueous solution (mg·L^−1^).

### 2.7. Calculation of the Adsorption Capacity of Toluene in Aqueous Solution

The adsorption capacity of toluene in aqueous solution can be calculated using the following equation:

Qt = (C_0_ − C_t_)V/m, (2)

where Qt is the adsorption capacity of toluene in aqueous solution (mg·g^−1^), C_0_ is the initial concentration of toluene in aqueous solution (mg·L^−1^), C_t_ is the concentration of residual toluene in the aqueous solution at time t (mg·L^−1^), V is the volume of toluene solution (mL), and m is the mass of adsorbents used (g).

When the adsorption of toluene in aqueous solution reaches equilibrium, Equation (2) can be expressed as follows:

Q_e_ = (C_0_ − C_e_)V/m, (3)

where Q_e_ is the equilibrium adsorption capacity of toluene in aqueous solution (mg·g^−1^), C_0_ is the initial concentration of toluene in aqueous solution (mg·L^−1^), C_e_ is the concentration of residual toluene in the aqueous solution when the adsorption reaches equilibrium (mg·L^−1^), V is the volume of toluene solution (mL), and m is the mass of adsorbents used (g).

## 3. Results and Discussion

### 3.1. Structural Characterization of Silico-Aluminate Nanoparticles

**Figure 1:**
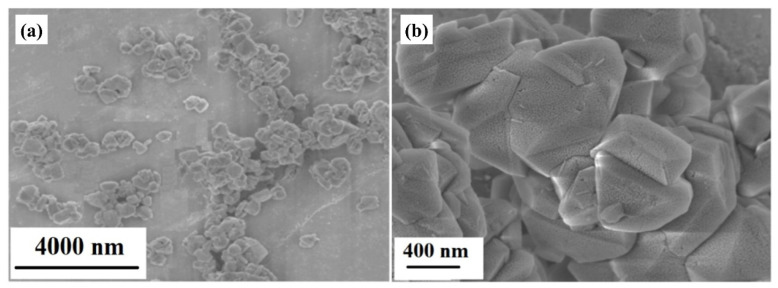
SEM images of the porous silico-aluminate nanoparticles under different magnifications: (**a**) 2000×; (**b**) 30,000×.

Figure 1 shows the SEM image of the as-synthesized porous silico-aluminate nanoparticles. The silico-aluminate nanoparticles are composed of many small nanoclusters stacked together. The silico-aluminate nanoparticles have a rough surface with a polyhedral structure and a porous sponge-like structure. The average particle size is approximately 500 nm.

**Figure 2:**
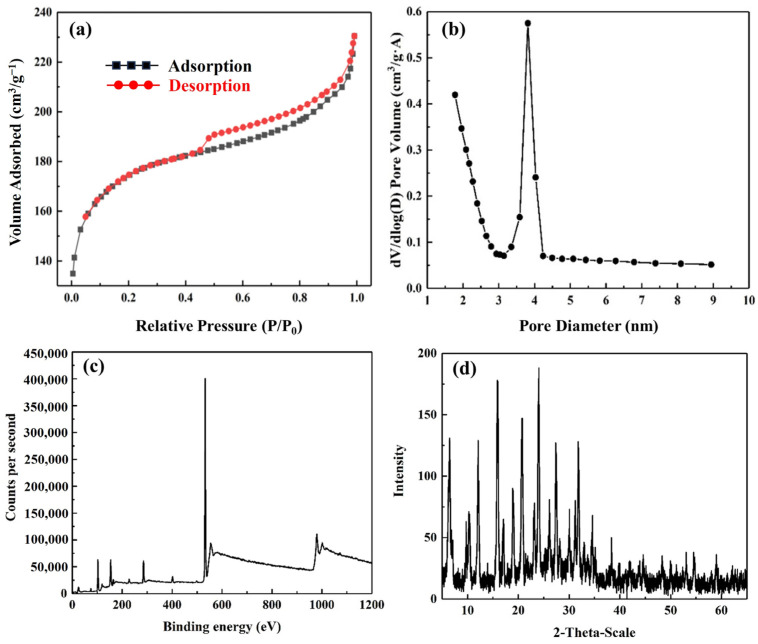
(**a**) N_2_ adsorption–desorption isotherms and (**b**) BJH desorption pore size distributions of the porous silico-aluminate nanoparticles; (**c**) Full-scan XPS profile of the as-synthesized porous silico-aluminate nanoparticles; (**d**) XRD pattern of the porous silico-aluminate nanoparticles.

Figure 2a shows the N_2_ adsorption–desorption isotherms of the as-synthesized porous silico-aluminate nanoparticles. The porous silico-aluminate nanoparticles exhibited typical Type-I and Type-IV isotherm characteristics. At relatively low pressures, the microporous filling mechanism plays a major role in the adsorption process, leading to a rapid increase in the adsorption capacity of porous silico-aluminate nanoparticles. Subsequently, the adsorption capacity of the porous silico-aluminate nanoparticles slowly increased with increasing relative pressure. In the relative pressure ranging from 0.43 to 0.98, a significant hysteresis loop appeared between the adsorption–desorption isotherms, indicating a strong capillary condensation phenomenon between N_2_ and the porous silico-aluminate nanoparticles. It can be inferred that porous silico-aluminate nanoparticles have multi-level pore structures, such as both micropores and mesopores. Figure 2b shows the pore size distribution of the porous silico-aluminate nanoparticles calculated using the BJH method based on the desorption isotherm. A significant mesoporous peak appeared at 3.1–4.2 nm, and the average pore size of the porous silico-aluminate nanoparticles was approximately 3.8 nm. At a relative pressure of 0.2272, the specific surface area calculated using the Brunauer–Emmett–Teller (BET) method was 582.08 cm^2^·g^−1^. At a relative pressure of 0.9695, the total pore volume of single-point adsorption was 0.33 cm^3^·g^−1^, the micropore volume was 0.19 cm^3^·g^−1^, and the mesopore volume was 0.14 cm^3^·g^−1^. Based on the results above, porous silico-aluminate nanoparticles have large specific surface areas and pore volumes.

Figure 2c shows the full-scan XPS profile of the as-synthesized porous silico-aluminate nanoparticles. There were several typical signals at binding energies of 74.58, 102.98, 284.78, 532.28, and 1072.58 eV, which were assigned to the characteristic peaks of Al2p, Si2p, C1s, O1s, and Na1s, respectively, indicating the presence of elements such as aluminum (Al), silicon (Si), carbon (C), oxygen (O), and sodium (Na) on the surface of the porous silico-aluminate nanoparticles. Table 1 shows the XPS elemental analysis results of the as-synthesized porous silico-aluminate nanoparticles. The porous silico-aluminate nanoparticles were mainly composed of SiO_2_ and Al_2_O_3_. The SiO_2_/Al_2_O_3_ ratio was approximately 3.3, indicating that the porous silico-aluminate nanoparticles showed significant characteristics of Y-shaped molecular sieves [34].

Figure 2d shows the XRD spectrum of the porous silico-aluminate nanoparticles. The X-ray diffraction patterns of the as-prepared porous silico-aluminate nanoparticles were consistent with those of typical NaY molecular sieves [35]. The diffraction peaks observed at 2θ = 6.2°, 10.1°, 11.9°, 15.7°, 18.7°, 20.4°, 23.7°, 27.1°, and 31.4° and were attributed to the FAU crystalline structure of the porous silico-aluminate nanoparticles. In addition, there was a wide diffraction peak at 2θ = 28° in the XRD pattern, indicating that there was a small amount of amorphous components in the synthesized powder. Overall, the X-ray diffraction peaks are relatively sharp, indicating that the as-prepared porous silico-aluminate nanoparticles had a good crystalline structure.

### 3.2. Effects of Different Dosages of Porous Silico-Aluminate Nanoparticles on the Removal Efficiency of Toluene

**Figure 3:**
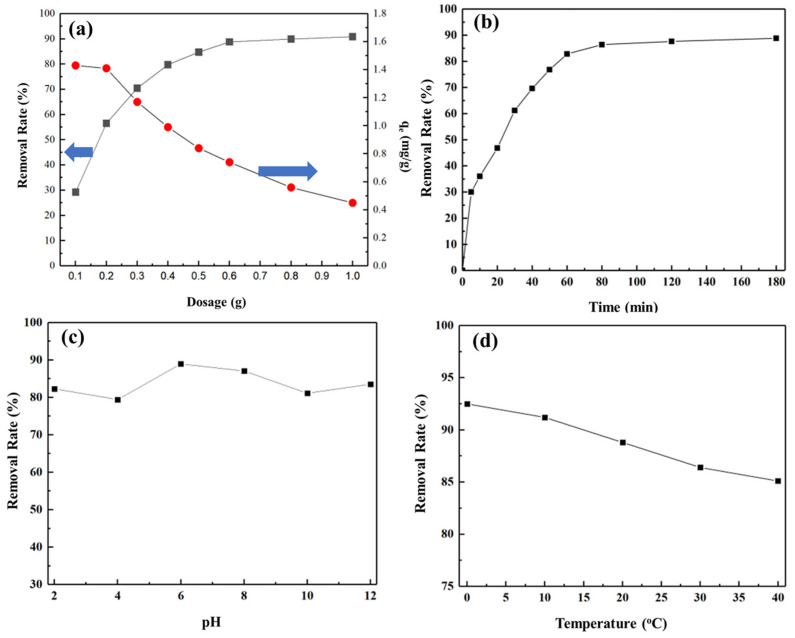
(**a**) Influence of the dosage of porous silico-aluminate nanoparticles on the removal rate of toluene; Effect of (**b**) capture time, (**c**) pH, and (**d**) temperature on the removal efficiency of toluene.

Figure 3a shows the effects of different dosages of porous silico-aluminate nanoparticles on the removal efficiency of toluene. With the increase in the amount of porous silico-aluminate nanoparticles, the removal rate of dissolved toluene in an aqueous solution tended to increase, while the equilibrium adsorption capacity of toluene tended to decrease. This was because the adsorption of porous silicon-aluminum to toluene occurs via physical adsorption, and as the dosage increased, the probability of toluene molecules coming into contact with the porous silico-aluminate nanoparticles increased, thus the removal rate increased. When the quantity of porous silico-aluminate nanoparticles used was 0.6 g, the removal rate of toluene reached 88.8%, and the increasing trend tended to be gradual. The decrease in the equilibrium adsorption capacity was attributed to the decrease in the probability of a unit quantity of porous silico-aluminate nanoparticles in aqueous solution coming into contact with toluene molecules as the dosage increased, resulting in a decrease in the equilibrium adsorption capacity. In addition, toluene molecules entered the internal pores and channels of porous silicate nanoparticles and blocked the first adsorption layer, which also led to a decrease in equilibrium adsorption. As the initial concentration of toluene in water decreases, the extraction rate of nanoparticles decreases sharply; this is also verified by the kinetics. These two curves intersected at approximately 0.3 g, indicating that the removal rates reached a relatively optimal value when the dosage was 0.3 g. The results showed that the porous silico-aluminate nanoparticles were highly efficient at eliminating toluene from aqueous solution.

### 3.3. Effect of the Capture Time on the Removal Efficiency of Toluene

Figure 3b shows the effect of capture time on the removal efficiency of toluene using porous silico-aluminate nanoparticles. The adsorption rate curve increased rapidly in the initial stage but subsequently slowed in the later stage. After 5 min, the adsorption rate of toluene in aqueous solution by the porous silico-aluminate nanoparticles reached 30%. After 80 min, the adsorption rate of toluene reached 86%. Then, as the reaction time increased, the adsorption rate increased very slowly. In summary, the adsorption of toluene by porous silico-aluminate nanoparticles is a rapid process that can reach equilibrium within 80 min.

### 3.4. Effect of pH on the Removal Efficiency of Toluene

Figure 3c shows the effect of pH on the removal efficiency of toluene by the porous silico-aluminate nanoparticles. Within the pH range from 2 to 12, the removal efficiency of toluene in aqueous solution by porous silico-aluminate nanoparticles shows a decreasing–increasing–decreasing–increasing trend. When the pH value ranged from 6 to 8, the porous silico-aluminate nanoparticles had a relatively high absorption rate for toluene in aqueous solution. Low selectivity is a major characteristic of physical adsorption. When the solution was acidic, the adsorption sites of the porous silico-aluminate nanoparticles were partly occupied by hydrogen ions rather than by toluene, resulting in a slight decrease in removal efficiency [36]. With increasing pH, hydrogen ions replaced some of the cations on the porous silico-aluminate nanoparticles to form silico-aluminate nanoparticles with electronic interactions. Due to the electron-donating properties of hydrogen atoms bound to carbon atoms in toluene molecules, their π orbitals provided electrons, which combined with porous silico-aluminate nanoparticles to form charge-transfer complexes, resulting in a certain increase in removal efficiency. Similarly, when the solution was alkaline, the adsorption sites of the porous silico-aluminate nanoparticles were partly occupied by hydroxide ions, resulting in a decrease in removal efficiency [37]. However, the hydrogen atoms in the hydroxide ions adsorbed on the surface of the porous silico-aluminate nanoparticles also had electron-acceptor interactions and formed charge-transfer complexes with toluene molecules that provided π-orbital electrons, resulting in an increase in removal efficiency. Therefore, when the pH of the solution was 6–8, the porous silico-aluminate nanoparticles effectively adsorbed toluene in aqueous solutions. An increase in the acidity or alkalinity of the solution was not conducive to removing toluene from aqueous solutions by porous silico-aluminate nanoparticles. However, at all pH values tested, the removal rate of toluene remained in the range of 80–90% and did not change much. Therefore, it can be considered that the adsorption process of toluene by nanomaterials is not significantly affected by the change in pH.

### 3.5. Effect of Temperature on the Removal Efficiency of Toluene

The effect of temperature on the removal efficiency of toluene using porous silico-aluminate nanoparticles is shown in Figure 3d. Over the whole temperature range investigated from 0 °C to 40 °C, the removal efficiency of toluene using porous silico-aluminate nanoparticles slightly decreased with increasing temperature. At relatively low temperatures, the adsorption rate of toluene increased, indicating that as the temperature increased, the number of active sites on the surface of the porous silico-aluminate nanoparticles weakened their ability to adsorb toluene molecules. The adsorption process may be an exothermic reaction [38]. This phenomenon conforms to the characteristics of physical adsorption. In addition, increasing temperature will increase the energy of toluene molecules, requiring stronger surface interactions for adsorption, and physical adsorption itself is an exothermic process, thus as the temperature increases, the adsorption amount and the removal rate decrease.

Table 2 shows the comparison between SANs and other nanomaterials reported in previous studies. The main variables included temperature, dosage of adsorbent, adsorption time, removal rate, etc. Through a comparison of related work, it could be seen that SANs could achieve a very high removal rate for toluene (>85%) in a short adsorption time with a certain amount of adsorbent at room temperature, which proved that SANs are an efficient toluene absorbent.

### 3.6. Adsorption Kinetic Study

**Figure 4:**
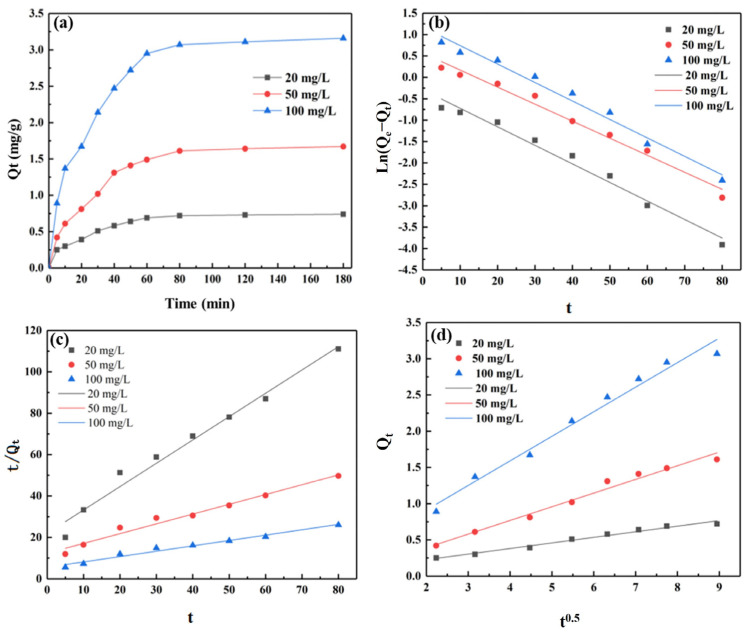
(**a**) Adsorption kinetics of different initial concentrations of toluene by porous silico-aluminate nanoparticles; The fitting results of the (**b**) pseudo-first-order, (**c**) pseudo-second-order and (**d**) Weber–Morris kinetics model for the absorption of toluene on porous silico-aluminate nanoparticles.

To reveal the adsorption mechanism, the kinetics of toluene adsorption by porous silico-aluminate nanoparticles in aqueous solutions were studied. Figure 4a shows the adsorption kinetic curves of different initial concentrations of toluene by the porous silico-aluminate nanoparticles. With increasing adsorption time, the initial adsorption capacity (Qt) of toluene in aqueous solution by the porous silico-aluminate nanoparticles increased rapidly and then decreased. The instantaneous adsorption rate (dQt/dt) gradually decreased until a plateau reached the adsorption equilibrium state when the adsorption capacity Qt increased. The curve can be divided into three stages. In the first stage (≤10 min), the slope was high, meaning that the adsorption capacity rapidly increased. Within 10 min, the adsorption capacity at equilibrium was approximately 40%, indicating that the adsorption of toluene by the porous silico-aluminate nanoparticles in this stage was rapid. Furthermore, the adsorption capacity increased with increasing initial concentration of toluene. In the second stage (from 10 min to 80 min), the slope slowly increased, meaning that the adsorption capacity increased relatively slowly with increasing adsorption time. In this stage, the adsorption equilibrium capacity was approximately 55%. In the third stage (>80 min), the slope was very low. In this stage, the adsorption rate hardly changed with increasing adsorption time, indicating that the porous silico-aluminate nanoparticles reached an adsorption equilibrium state. Therefore, the adsorption of toluene by the porous silico-aluminate nanoparticles was relatively fast, and the amount absorbed at equilibrium increased with increasing initial concentration of toluene.

The kinetics of toluene adsorption on porous silico-aluminate nanoparticles were studied using the pseudo-first-order kinetics model, pseudo-second-order kinetics model, and Weber–Morris kinetics model.

#### 3.6.1. The Pseudo-First-Order Kinetics

The formula for pseudo-first-order dynamics is shown below (4). The fitting results of the pseudo-first-order kinetics model for the absorption of toluene on the porous silico-aluminate nanoparticles are displayed in Figure 4b, and the kinetic parameters are listed in Table 3. The Karl Pearson correlation coefficients obtained through linear fitting of the experimental data are relatively high, with R values greater than 0.99, indicating a good linear correlation between ln(Qe − Qt) and t. In addition, the relative error between the theoretical and experimental values of the adsorption rate of toluene by the porous silico-aluminate nanoparticles was relatively low, indicating that the Qe value calculated by the model was very close to the measured value. Therefore, the pseudo-first-order kinetics model can accurately describe the process of toluene adsorption by the porous silico-aluminate nanoparticles, indicating that the adsorption process was controlled by the diffusion of toluene molecules to the outer surface of the porous silico-aluminate nanoparticles [43].

(4) $$\ln\left(Q_{e}-Q_{t}\right)=\ln Q_{e}-K_{1}t$$

#### 3.6.2. The Pseudo-Second-Order Kinetics

The formula for the pseudo-second-order dynamics is shown below (5). The fitting results of the pseudo-second-order kinetics model for the absorption of toluene on the porous silico-aluminate nanoparticles are shown in Figure 4c, and the kinetic parameters are summarized in Table 4. All the Karl Pearson correlation coefficient (R) values obtained through linear fitting of the experimental data were lower than 0.99, indicating that the linear correlation between t/Qt and t was relatively poor. In addition, there was a significant relative error between the theoretical and experimental values of the adsorption capacity at the equilibrium of toluene by the porous silico-aluminate nanoparticles, indicating that the Qe value calculated by this model deviated significantly from the measured value. Therefore, compared with the pseudo-one-order kinetics model, the pseudo-second-order kinetics model was not suitable for describing the adsorption process of toluene by porous silico-aluminate nanoparticles. These results indicated that chemical adsorption was not a limiting factor in the adsorption process and that toluene molecules did not bind to the porous silico-aluminate nanoparticles by sharing electrons.

(5) $$\frac{t}{Q_{t}}=\frac{t}{Q_{e}}+\frac{1}{K_{2}Q_{e}^{2}}$$

#### 3.6.3. Weber–Morris Kinetics

The formula for the Weber–Morris kinetics is shown below (6). The fitting results of the Weber–Morris kinetics model for the absorption of toluene on the porous silico-aluminate nanoparticles are shown in Figure 4d, and the kinetic parameters are summarized in Table 5. The Karl Pearson correlation coefficients obtained through linear fitting of the experimental data were relatively high, with R values greater than 0.99, indicating a good linear correlation between Qt and t^0.5^. The results suggested that the Weber–Morris kinetic model can accurately describe the process of toluene adsorption by the porous silico-aluminate nanoparticles. In addition, the fitting line of the Weber–Morris kinetic diffusion equation did not cross the origin, implying that the adsorption process was mainly controlled by intra-particle diffusion [44].

Q_t_ = K_i_t^0.5^ + C (6)

Based on the analysis above, all these models can describe the adsorption process of toluene on porous silicon aluminate nanoparticles. Compared with the pseudo-second-order kinetics model, the pseudo-one-order kinetics model and Weber–Morris kinetics model can better describe the adsorption process of toluene on porous silico-aluminate nanoparticles. It can be inferred that chemical adsorption was not a limiting step in the adsorption process. The binding between toluene and the porous silico-aluminate nanoparticles did not occur through the sharing of electrons but rather through intermolecular electrostatic forces. The process of toluene adsorption by the porous silico-aluminate nanoparticles was mainly controlled by intra-particle diffusion, but intra-particle diffusion was not the only rate-limiting step.

## 4. Conclusions

In this paper, porous silico-aluminate nanoparticles (SANs) were prepared from cost-effective and non-toxic natural kaolin using a hydrothermal method. The morphology and composition of the SANs were confirmed using SEM, XPS, XRD, and BET-specific surface area tests. The SANs showed a polyhedron morphology containing hierarchical pore structures. The average particle size was approximately 500 nm, the average pore diameter was approximately 3.8 nm, and the specific surface area was as high as 582.08 cm^2^·g^−1^. The SANs exhibited the remarkable structural composition characteristics of Y-type molecular sieves with a molar ratio of SiO_2_ to Al_2_O_3_ of 3.3:1. The adsorption of toluene by porous SANs was a fast process, reaching an equilibrium state within 80 min. When the pH of the solution ranged from 6 to 8, the porous SANs effectively adsorbed toluene. With increasing temperature from 0 °C to 40 °C, the adsorption rate slightly decreased because the adsorption effect of the active sites on the surface of the porous SANs was weakened. Adsorption kinetics analysis revealed that the adsorption of toluene by porous SANs was mainly controlled by intra-particle diffusion.

## Figures and Tables

**Table 1:** Chemical composition of the porous silico-aluminate nanoparticles.

Atom	O	Si	C	Al	Na
Atomic conc. (%)	55.0	21.7	19.7	3.3	0.3

**Table 2:** Comparison of the adsorption capacity between SANs and other nanomaterials reported in previous studies.

Sorbent Name	Temperature (°C)	Dosage (g)	Adsorption Time	Removal Rate (%)	References
Zeolite Na-P1	20	0.5	24 h	<60	[39]
Zeolite SMZ-100	20	1.5	24 h	<80	[40]
Zeolite–hemp composite	25	--	300 min	<80	[41]
Fe-Al/B	80	1.0	120 min	<70	[42]
SANs	20	0.6	80 min	86	This Work

**Table 3:** The pseudo-first-order kinetics equations and parameters for the absorption of different initial concentrations of toluene on porous silico-aluminate nanoparticles.

Initial Concentration C_0_ (mg·L^−1^)	Fitting Equation	Correlation Coefficient (R)	R^2^	Rate Constant k_1_ (min^−1^)	Adsorption Capacity at Equilibrium Q_e_ (mg·g^−1^)
20	ln(Q_e_ − Q_t_) = −0.28951 − 0.04333t	0.99022	0.9805	0.04333	0.7486
50	ln(Q_e_ − Q_t_) = 0.5683 − 0.03981t	0.99107	0.9822	0.03981	1.7653
100	ln(Q_e_ − Q_t_) = 1.1736 − 0.04315t	0.99034	0.9808	0.04315	3.2336

**Table 4:** The pseudo-second-order kinetics equations and parameters for the absorption of different initial concentrations of toluene on porous silico-aluminate nanoparticles.

Initial Concentration C_0_ (mg·L^−1^)	Fitting Equation	Correlation Coefficient (R)	R^2^	Rate Constant k_2_ [g·(mg·min)^−1^]	Adsorption Capacity at Equilibrium Q_e_ (mg·g^−1^)
20	t/Q_t_ = 21.9904 + 1.1277t	0.98962	0.97935	0.05782	0.8868
50	t/Q_t_ = 12.3681 + 0.4726t	0.98760	0.97535	0.01806	2.1161
100	t/Q_t_ = 5.52834 + 0.2595t	0.98894	0.97800	0.01218	3.8530

**Table 5:** The Weber–Morris kinetics equations and parameters for the absorption of different initial concentrations of toluene on porous silico-aluminate nanoparticles.

Initial Concentration C_0_ (mg·L^−1^)	Fitting Equation	Correlation Coefficient (R)	R^2^	Rate Constant K_pi_ [g·(mg·min^0.5^)^−1^]	Diffusion Constant C
20	Q_t_ = 0.07715t^0.5^ + 0.07188	0.99028	0.98065	0.07715	0.07188
50	Q_t_ = 0.18876t^0.5^ + 0.01303	0.99019	0.98048	0.18876	0.01303
100	Q_t_ = 0.33916t^0.5^ + 0.23387	0.99002	0.98014	0.33916	0.23387

## Data Availability

The data presented in this study are available on request from the corresponding author. The data are not publicly available due to specific ethical and privacy considerations.
